# Combined Cryoanalgesia and Erector Spinae Plane Block for Postoperative Pain Management in the Nuss Procedure: A Case Report

**DOI:** 10.7759/cureus.98912

**Published:** 2025-12-10

**Authors:** Filipa Rosa, Marta Marques, Sara Lopes, Inês Correia, Amélia Ferreira

**Affiliations:** 1 Anesthesiology, Unidade Local de Saúde de São João, Porto, PRT

**Keywords:** cryoablation, erector spinae plane block, multimodal analgesia, nuss procedure, pectus excavatum

## Abstract

Pectus excavatum, the most common congenital chest wall deformity, is conventionally corrected using the minimally invasive Nuss procedure; however, postoperative pain remains a significant clinical challenge. Thoracic epidural analgesia (TEA) has traditionally been considered the gold standard, but it carries potential complications and technical challenges, particularly in patients with thoracic deformities. Alternative strategies, including the erector spinae plane block (ESPB) and intercostal nerve cryoablation, are therefore being explored to optimize pain control, reduce opioid consumption, and facilitate recovery. Intercostal nerve cryoablation provides prolonged analgesia through temporary axonal degeneration, yet its delayed onset necessitates complementary techniques for effective immediate postoperative pain management.

We report the case of a 14-year-old female (American Society of Anesthesiologists Physical Status III (ASA III)) with significant thoracic deformity who underwent elective correction of pectus excavatum using the Nuss procedure. After induction of general anesthesia, bilateral single-shot pre-incision ESPB were performed at the T5 level with 0.375% ropivacaine. Thoracoscopic intercostal nerve cryoablation (T3-T7) was then performed under selective lung isolation using a CRYO-S Painless cryoprobe (Metrum Cryoflex, Łomianki, Poland) with high-pressure carbon dioxide, applied directly to the intercostal nerves bilaterally. Each nerve received a single 2-minute cycle reaching −70 °C, followed by bar placement. Postoperative multimodal analgesia included paracetamol, ketorolac, magnesium sulfate, ketamine, and tramadol. Upon arrival in the post-anesthesia care unit, the patient reported a Visual Analog Scale (VAS) score of 8/10, described as thoracic heaviness/pressure, although her clinical presentation did not clearly suggest pain of this intensity. For additional comfort, an intravenous tramadol-droperidol infusion was administered for approximately 3 hours, reducing pain to VAS 3/10. On postoperative day 1, VAS peaked at 2/10 and decreased to 0/10 thereafter. The patient was discharged on postoperative day 3 with well-controlled pain, and no complications were reported.

This combined approach appeared to provide effective immediate and sustained analgesia, minimize opioid use, and facilitate early mobilization and recovery. ESPB ensured rapid thoracic wall analgesia, while cryoablation offered prolonged pain control. Compared with TEA or paravertebral blocks, this strategy may offer comparable efficacy with potential advantages in safety and technical simplicity. The integration of ESPB with intercostal nerve cryoablation could represent a promising multimodal analgesic approach, supporting enhanced recovery and warranting further evaluation for broader application in perioperative pain management.

## Introduction

Pectus excavatum is the most common congenital chest wall deformity, affecting approximately 6.3-12 per 1,000 individuals worldwide [1]. The Nuss procedure has become the standard of care, involving the placement and rotation of metal bars to correct the chest wall deformity [2]. Despite its minimally invasive nature, this surgery is associated with significant postoperative pain, which often requires high opioid doses, contributing to prolonged hospitalization [1]. Effective pain control remains a key factor influencing hospital length of stay after the Nuss procedure; Therefore, optimizing postoperative analgesia is a crucial aspect of patient care in this surgical approach [3].

Traditionally, postoperative analgesia relied on a combination of opioids under patient-controlled analgesia (PCA) regimens, continuous regional nerve blocks, and thoracic epidural catheters [4]. Thoracic epidural analgesia (TEA) has been considered the gold standard for pain management in this surgery; however, this technique carries potential risks, including hypotension, urinary retention, and, in rare cases, epidural hematoma or infection. Furthermore, the placement of a thoracic epidural catheter can be technically challenging, particularly in pediatric and adolescent patients, requiring close postoperative monitoring [1,5,6].

To address these limitations, alternative strategies are being explored, including intercostal nerve cryoablation. This technique employs a commercially available cryoprobe that delivers high-pressure carbon dioxide (CO₂) or nitrous oxide, rapidly cooling the targeted nerves to approximately −50 to −70 °C. Such temperatures induce Wallerian degeneration of the axons, transiently interrupting nociceptive signaling, while preserving the external connective layers, which allows full axonal regeneration within 4-6 weeks [1]. Cryoablation has demonstrated substantial efficacy in reducing postoperative opioid requirements [3,7]; however, because its complete analgesic effect develops only after 24 hours, supplementary strategies are essential to ensure adequate pain control until the full effect is achieved [1,3]. In this context, combining cryoablation with the erector spinae plane block (ESPB) appears particularly promising [2].

In this report, we describe the combined use of bilateral single-shot pre-incision ESPB with thoracoscopic intercostal nerve cryoablation in a patient undergoing the Nuss procedure, discussing the benefits and challenges of this approach. Written informed consent was obtained from the patient and her legal representatives for publication of this case report and complementary images.

## Case presentation

**Figure 1:**
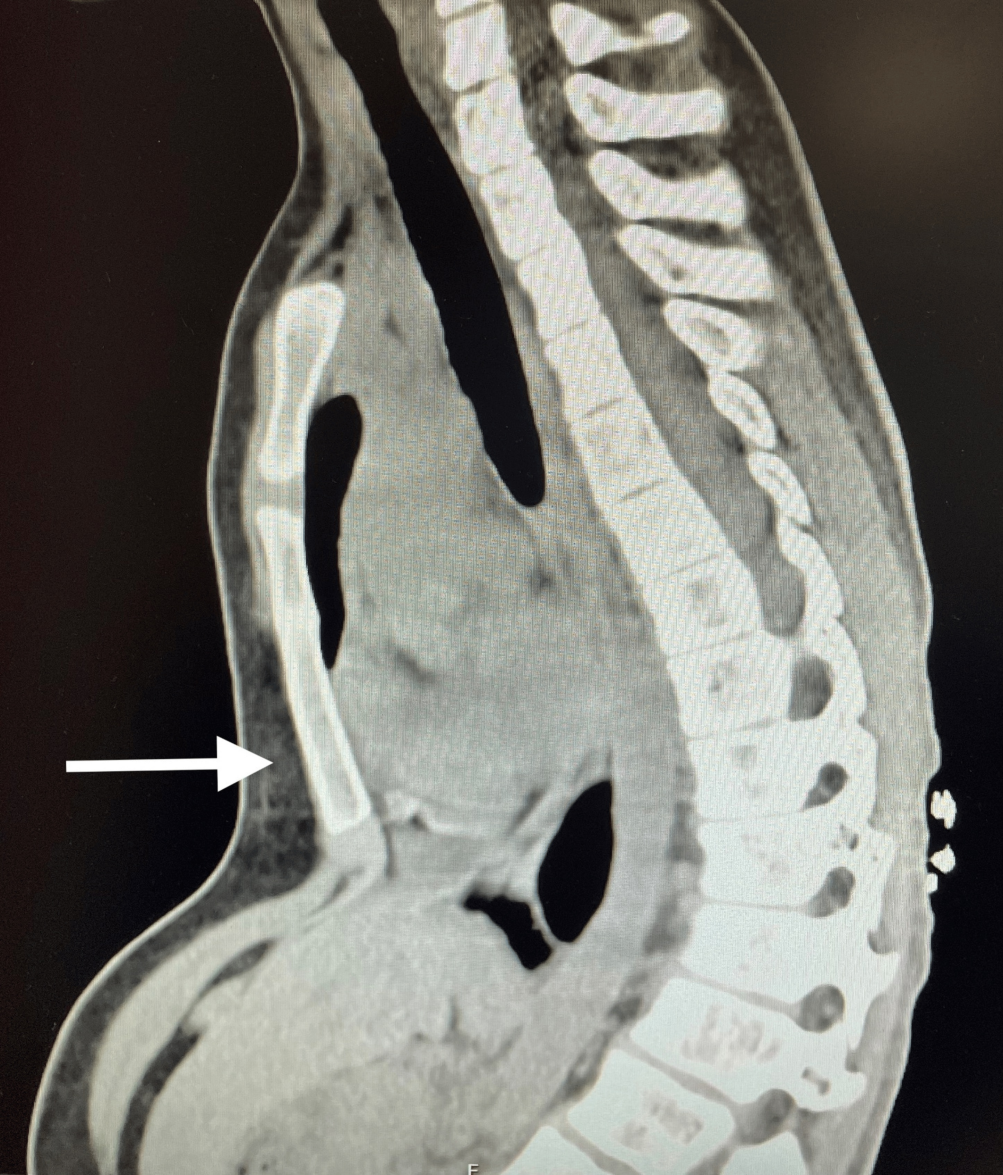
Computed tomography (CT) image demonstrating the thoracic deformity (arrow) consistent with pectus excavatum.

A 14-year-old female weighing 57 kg, classified as American Society of Anesthesiologists (ASA) Physical Status III, was admitted for elective pectus excavatum correction (Haller index of 3.4) using the Nuss procedure (Figure 1). Her medical history was notable for scoliosis and a localized epidural Ewing's sarcoma, treated with laminectomy (T10-T11 level), as well as radiotherapy and chemotherapy throughout 2019 and 2020, which ultimately led to a significant thoracic spinal deformity. Preoperative laboratory findings were normal, transthoracic echocardiography revealed no structural abnormalities, and the electrocardiogram performed showed sinus rhythm. Previous surgical interventions were performed without any anesthetic complications, and no difficulties in airway management were anticipated.

The patient was monitored according to the ASA Standards for Basic Anesthetic Monitoring (Standards I and II), and a processed electroencephalogram using the Bispectral Index (BIS, Medtronic, Minneapolis, MN) system was employed. After establishing a venous catheter, general anesthesia was induced with intravenous boluses of fentanyl (3 mcg/kg), lidocaine (1 mg/kg), propofol (3 mg/kg), and rocuronium bromide (1.2 mg/kg). Orotracheal intubation was uneventfully performed using direct laryngoscopy and a 35 Fr left double-lumen tube, and correct positioning for selective lung ventilation was confirmed by fiberoptic bronchoscopy. Two large-bore venous accesses were secured, and anesthesia was maintained using sevoflurane and rocuronium bromide administered in boluses as needed.

Before surgical incision, a bilateral ESPB was performed under ultrasound guidance at the T5 level using a 22-G 50 mm PAJUNK needle (PAJUNK GmbH, Geisingen, Germany), with a total of 40 mL of 0.375% ropivacaine (20 mL per side). The surgical procedure began with cryoablation of the intercostal nerves from T3 to T7, performed by the surgical team via video-assisted thoracoscopy. A CRYO-S® Painless cryoprobe (A30/PEA/R/RF; length 425 mm, diameter 3 mm, 35° angulation; Metrum Cryoflex, Łomianki, Poland) using high-pressure CO₂ was applied directly to the intercostal nerves posterolateral to the chest wall incisions, targeting the nerves at the level of the incision as well as the two interspaces above and below, bilaterally. Each nerve received a single 2-minute cycle of cryoablation, reaching a temperature of −70 °C, with selective lung isolation alternated as needed to facilitate the procedure. Subsequently, the Nuss procedure was initiated with the placement of a thoracic bar under video-assisted thoracoscopy surgery (VATS). The surgery was uneventful, with minimal blood loss.

As part of a multimodal analgesic strategy, systemic analgesia included paracetamol 850 mg, ketorolac 30 mg, magnesium sulfate 2,000 mg, ketamine 20 mg, and tramadol 100 mg. Postoperative nausea and vomiting prophylaxis was provided with 4 mg of ondansetron and 8 mg of dexamethasone, to provide antiemetic and anti-inflammatory effects as well as to prolong the analgesic effect of the regional technique. The patient was extubated uneventfully and transferred to the post-anesthesia care unit (PACU).

The postoperative analgesia strategy included paracetamol (15 mg/kg every 6 hours), ketorolac (0.5 mg/kg every 8 hours), and morphine (0.1 mg/kg) as rescue. On postoperative day 0, upon arrival in the PACU, the patient reported a maximum pain score of 8/10 on the Visual Analog Scale (VAS), describing a sensation of thoracic heaviness/pressure, although she did not appear to exhibit physiological or behavioral signs consistent with this score. For additional comfort, an intravenous infusion of tramadol (600 mg) combined with droperidol (2.5 mg), diluted to a final volume of 100 mL, was administered at 2 mL/hour (12 mg/hour) and discontinued after approximately 3 hours once pain was adequately controlled (VAS 3/10), before transfer to the ward. On postoperative day 1, pain intensity further decreased to a peak VAS score of 2/10, without the need for rescue analgesia, and on postoperative days 2 and 3, the patient remained comfortable and reported no pain (VAS 0/10). No rescue morphine was required throughout the hospital stay, and the patient was discharged on postoperative day 3 with well-controlled pain and without any reported complications.

## Discussion

Although minimally invasive, the Nuss procedure is associated with substantial postoperative pain [1,4]. While there is no widely agreed-upon approach to pain relief, the trend towards multimodal analgesia is gaining traction. Numerous studies and clinical reports demonstrate the effectiveness of various techniques, including opioids under PCA regimens, thoracic epidural catheters, paravertebral blocks (PVBs), single-shot or continuous ESPB, as well as intercostal nerve blocks or ablations [4,5,8]. No single technique has been proven superior across all settings, and institutional and patient-specific factors often guide practice [5].

Cryoablation has gained attention as an effective modality for managing post-Nuss procedure pain. This technique uses extreme cold (-50 to -70 ºC) to disrupt peripheral nerve conduction through Wallerian degeneration, which temporarily blocks pain transmission while preserving the outer neural structures, allowing complete axonal regeneration within four to six weeks [1,7,9-11]. In the context of pectus excavatum repair, intercostal nerve cryoablation is particularly advantageous as it directly targets the primary pain pathways involved. Thoracoscopic guidance following selective lung isolation allows precise identification of the intercostal nerves, optimizing the effectiveness of the technique. The existing literature, including the randomized clinical trial by Graves et al. [1], has demonstrated that intercostal nerve cryoablation significantly reduces opioid requirements and accelerates recovery, due to its effective and prolonged analgesic effect, leading to an earlier hospital discharge. Although the exact onset time of analgesia from intercostal nerve cryoablation remains uncertain, studies suggest that patients typically start experiencing its effects within the first 24 hours [1,3,5]. This implies that cryoablation alone may not be sufficient to provide adequate pain relief in the immediate postoperative period, highlighting the importance of integrating cryoablation into a multimodal pain management approach to address this limitation [2,5].

Combining cryoablation with other pain management techniques, such as regional blocks, has been shown to improve its effectiveness, providing optimal analgesia during both the intraoperative and postoperative periods. Although TEA has traditionally been considered the gold standard for pain control, the single-shot ESPB has emerged as a promising alternative due to its efficacy, simplicity, and lower risk profile. The ESPB is a regional anesthesia technique that targets the erector spinae muscle, which runs along the spine. It involves placing the needle between the erector spinae muscle and the thoracic transverse processes to administer a local anesthetic. This blocks both the dorsal and ventral rami of the thoracic and abdominal spinal nerves, resulting in a multi-dermatomal sensory block that covers the anterior, posterior, and lateral thoracoabdominal wall [12]. Somatic and visceral analgesia is further enhanced by the spread of local anesthetic into the paravertebral space through the costotransverse foramina and the intertransverse complex. This diffusion contributes to effective pain control, being particularly beneficial for this type of surgery [6,12].

In this case, the ESPB was selected as an alternative to TEA due to the patient’s thoracic deformity, which could have rendered TEA technically challenging and associated with an increased risk of perioperative complications. Compared to TEA, ESPB is simpler to perform and avoids complications such as hypotension due to sympathetic blockade, urinary retention, and, in rare cases, epidural hematoma or infection. It is also easier and safer than PVB, with a lower risk of pneumothorax, vascular injury, or inadvertent epidural or spinal anesthesia. Additionally, when compared with opioid-based PCA regimens, ESPB minimizes systemic opioid-related side effects, including nausea, vomiting, ileus, sedation, and respiratory depression, which can delay recovery and prolong hospital stay [4].

Both cryoablation and ESPB are generally considered safe when performed by skilled practitioners. However, as with any intervention, they are not exempt from potential risks. Cryoablation, while effective for long-lasting pain relief, may cause transient sensory loss or dysesthesia in the treated dermatomes. These effects are typically self-limited, as axonal regeneration occurs within four to six weeks, and current evidence does not associate cryoablation with long-term neuropathic pain and permanent sensory deficits [1,9-10]. Nevertheless, improper probe position or excessive freezing time could theoretically damage surrounding tissues, highlighting the importance of thoracoscopic guidance, selective lung isolation, and meticulous intraoperative monitoring. 

The ESPB also has a favorable safety profile. It is performed under ultrasound guidance, which significantly reduces the risks of pleural puncture, pneumothorax, or vascular injury. Compared with TEA or PVB, ESPB is technically simpler, does not cause sympathetic block or significant hemodynamic instability, and is therefore particularly advantageous in pediatric and adolescent patients undergoing the Nuss procedure, aligning well with Enhanced Recovery After Surgery (ERAS) principles for thoracic surgery [2,6,12]. When both techniques are combined, their effects appear complementary rather than additive in risk. Nevertheless, close postoperative monitoring remains essential to identify rare complications early and optimize recovery [1,2].

Combining intercostal nerve cryoablation with bilateral single-shot ESPB before incision has shown promising results in improving postoperative pain control for patients undergoing the Nuss procedure. This multimodal approach allows ESPB to provide immediate thoracic analgesia while cryoablation takes effect, ensuring a seamless transition toward prolonged pain relief. Aranda-Valderrama et al. demonstrated that this strategy significantly reduces perioperative opioid consumption and facilitates earlier mobilization and discharge [2]. By minimizing opioid-related side effects, this technique enhances patient comfort and recovery, improves hospital workflow, and aligns with modern multimodal analgesia principles, representing a safe, effective, and opioid-sparing approach for postoperative pain management after the Nuss procedure [2].

In our patient, the ESPB appeared to play a key role in managing pain both during surgery and in the immediate postoperative period; however, it did not fully alleviate her sensation of thoracic pressure, with a patient-reported VAS score of 8/10 upon arrival in the PACU, although clinical signs suggested only moderate discomfort. Despite this isolated high score, several clinical observations support the effectiveness of the block. During the intraoperative period, she required no additional opioid boluses beyond induction and maintained stable hemodynamics throughout the procedure. Upon arrival in the PACU, her facial expression and overall presentation were not consistent with the reported VAS score, and she exhibited no physiological signs typically associated with significant pain, such as hypertension or tachycardia. Rapid analgesic improvement was achieved (VAS 3/10) with a low dose of intravenous opioids over a short period, even before the full onset of the cryoanalgesic effect. These findings suggest that the ESPB was likely effective and that the initially reported VAS may have reflected a subjective perception of thoracic pressure rather than true nociceptive pain. More detailed preoperative education regarding expected postoperative sensations might have further aligned patient-reported pain scores with objective clinical observations.

Despite these encouraging outcomes, current evidence supporting the combined use of intercostal nerve cryoablation and ESPB in the Nuss procedure remains limited to case reports and small observational studies. High-quality prospective and randomized clinical trials are still required to validate these findings and inform standardized protocols for their integration into routine anesthetic practice.

## Conclusions

The combination of thoracoscopic intercostal nerve cryoablation with ESPB appears to be a safe and effective analgesic strategy for postoperative pain management following the Nuss procedure. This approach ensured adequate immediate analgesia through ESPB while providing prolonged pain relief from cryoablation, resulting in minimal opioid consumption, early mobilization, and timely discharge. Given its favorable safety profile, technical simplicity, and compatibility with ERAS protocols, this multimodal strategy represents a valuable alternative to traditional techniques such as TEA or PVB in pediatric and adolescent patients undergoing the Nuss procedure. Further evaluation and experience may facilitate the refinement of its application and support its integration into standard perioperative care.
